# Towards a definition of microglia heterogeneity

**DOI:** 10.1038/s42003-022-04081-6

**Published:** 2022-10-20

**Authors:** Luke M. Healy, Sameera Zia, Jason R. Plemel

**Affiliations:** 1 Neuroimmunology Unit, Department of Neurology and Neurosurgery, Montreal Neurological Institute and Hospital, McGill University, Montreal, QC Canada; 2 Neuroscience and Mental Health Institute, University of Alberta, Edmonton, AB Canada; 3 Department of Medicine, Division of Neurology, University of Alberta, Edmonton, AB Canada; 4 Department of Medical Microbiology & Immunology, University of Alberta, Edmonton, AB Canada

**Keywords:** Microglia, Transcriptomics

## Abstract

High dimensional single-cell analysis such as single cell and single nucleus RNA sequencing (sc/snRNAseq) are currently being widely applied to explore microglia diversity. The use of sc/snRNAseq provides a powerful and unbiased approach to deconvolve heterogeneous cellular populations. However, sc/snRNAseq and analyses pipelines are designed to find heterogeneity. Indeed, cellular heterogeneity is often the most frequently reported finding. In this Perspective, we consider the ubiquitous concept of heterogeneity focusing on its application to microglia research and its influence on the field of neuroimmunology. We suggest that a clear understanding of the semantic and biological implications of microglia heterogeneity is essential for mitigating confusion among researchers.

## Abstract (web summary)

Microglia “heterogeneity” is often described in the literature, but a clear understanding of what “heterogeneity” entails is essential to avoid confusion among researchers.

## Introduction

Derived from the Greek ἕτερος (heteros; other, different) and γένος (genos; kind, gender), heterogeneity refers to the quality of uniformity (or lack thereof) in a substance, population, or mixture. A heterogeneous population is one made up of contrasting or diverse elements. Studies of heterogeneity are sensitive to several key parameters, these include scale, sample size, sampling strategy and magnitude.

While an object might appear homogenous on a macroscopic scale, heterogeneity can be revealed when viewed through the lens of sequentially more powerful optics. For example, metal surfaces can appear smooth to the naked eye but when subjected to electron microscopic investigation can reveal complex structural heterogeneity. Similarly, sample size (*n*) can have a considerable effect on one’s ability to detect heterogeneity. Typically, the larger the *n* number the more likely it is one will detect heterogeneity. This is due to a significant increase in the probability of observing rarer subpopulations or subsets within a large population. However, this may not always be the case. If one considers a bag of skittles candy, sampling by hand on separate occasions will reveal different combinations of numbers and colours of skittles. However, if one empties this bag into a bowl and compare the colours and their ratios this sampling heterogeneity is lost, and the true number of skittles is revealed in the bag. Therefore, choosing an appropriately sized sample and sampling enough times to generate an accurate picture is crucial to avoid the premature drawing of conclusions regarding the extent of heterogeneity of the bag. Finally, the precise point at which a population becomes heterogeneous remains to be defined by the investigator. For example, consider a bag of 100 marbles, all different colours and sizes, this is clearly a heterogeneous population. However, a bag of 99 black marbles of uniform size, plus one large red marble could also be described as heterogeneous. While both these bags can be described as heterogeneous, there is a clear distinction in magnitude. Drawing from this metaphor, a small population of transcriptionally distinct microglia responding uniformly to a focal point of injury and multiple distinct and diverse microglial subpopulations arising during development, would both be referred to as heterogeneous. A given field of investigation must be cognizant of the fact that ‘heterogeneity’ can encompass a range of complexity and overreliance on this word can lead to loss of important information and misunderstanding between researchers. If one considers heterogeneity to be a quality of immense importance, then a precise metric or heterogeneity ‘index’ may be useful to allow for the cross-sectional study of this phenomenon.

Ultimately, in the microglia field, the word ‘heterogeneity’ typically provokes interest as the word itself carries with it an inference of microglial functional diversity. It is this functional diversity that should expedite our understanding of diverse homoeostatic cellular functions and by extension, our understanding of diverse cellular responses to injurious and pathological microglial states.

## Microglia heterogeneity

Microglia are CNS resident immune cells that regulate the response to injury and disease, while also serving critical functions throughout development and life such as synaptic pruning and circuitry remodelling^1,2^. Microglia change their cellular state (see Box 1 for definitions), defined often by protein or gene-expression patterns, throughout development and during disease^3,4^. Microglia express >1000 receptor systems, of which >100 are uniquely expressed compared to other non-myeloid central nervous system (CNS) cells^5^, making them exquisitely sensitive to the extracellular environment. Perhaps, owing to this high responsivity, environmental conditions alter microglia to adopt diverse cellular states. However, microglia also originate from different sources including both yolk sac and early foetal monocytes^1^, which may also impart distinct responses to environmental signals. During development, microglia adopt distinct cellular states as indicated by the unique expression of one or more genes. For example, within the developing white matter tracts there is a microglial state enriched for genes such as Secreted phosphoprotein 1(*Spp1)*, Glycoprotein Nonmetastatic Melanoma Protine B(*Gpnmb)*, and Insulin-like Growth Factor (*Igf1)*^6,7^. Other proliferative, metabolically active, and phagocytic microglial states are present in development, but many of these diverse phenotypes are sparsely present after puberty in mice^6–9^.

During injury and disease, microglia again adopt a diverse range of cellular states. Along with the phenotypically similar myeloid cells such monocyte-derived macrophages, CNS meningeal, and perivascular macrophages, these cells have subtle, complex, and incompletely understood roles. For example, microglia and macrophages regulate myelin regeneration^10–13^ and promote neonatal axonal regeneration^14^ in the white matter. However, the roles of these cells are complex as they also drive autoimmune neurotoxicity and demyelination^15^. It is still unclear how microglia contribute to these divergent outcomes that propel or impede pathogenesis. However, it is tempting to speculate that the divergent regenerative or neurotoxic attributes of microglia relate to subpopulations of cells with functionally divergent properties, or cellular phenotypes. Simply put—and of great importance—some microglia phenotypes may be neurotoxic, while other phenotypes may support regeneration. Historically, microglia were assumed, based on their similarity to macrophages, to take on proinflammatory M1 or immunoregulatory M2 phenotypes, which may account for neurotoxic or regenerative phenotypes. However, using scRNAseq it becomes clear that classic M1 and M2 states are not identified in microglia in vivo^6,16,17^. Therefore, the properties of any putative regenerative or cytotoxic microglial states remain to be determined. A clear understanding of these cells is of great significance for the potential treatment of neurological conditions.

Heterogeneity in the microglia population is not altogether surprising. During microglia homoeostasis there is no evidence of the kind of clonal expansion that drives the phenotypic restriction seen in populations of lymphocytes. Instead microglia undergo slow, stochastic self-renewal^1^. Over time, microglia are exposed to their own unique microenvironment, which ultimately leads to the acquisition of distinctive transcriptional signatures^18–20^. As one considers microglia heterogeneity, it is also important to consider changes over time. A fundamental feature of microglia is their ability to rapidly sense, integrate and respond to signals from within the CNS compartment and from the periphery. The inclusion of longitudinal studies of microglia (and associated heterogeneity) will be important in distinguishing between how microglia respond to changes in diet, circadian rhythms, hormonal cycles, and other environmental factors.

Box 1. Microglia heterogeneity definitions How does the brain function during development, throughout life, and during disease or injury? These are fundamental questions that encapsulate much of neuroscience research. Historically, cell function was studied by taking a cell of interest, homogenizing the sample, and measuring either the protein or mRNA content to generate a ‘signature’. By combining this signature with specific gain/loss of function experiments, overall functions were ascribed to that cell. What we know now is that there is more diversity in the CNS, even within a particular cell type. With the dawn of the single-cell era, new cell populations are being discovered and understanding this cellular heterogeneity will be a focus for researchers in the decades to come. For this review, we refer to a unique cellular signature based on protein or mRNA markers as a cell *state*. For high dimensional data, often projected onto a UMAP or TSNE, data is sorted through an algorithm and organized into one or more *clusters*. If cell state is defined based on clusters from UMAP or TSNE data, others may also refer to this as a *subpopulation*, *subtype*, or *subset*. However, a recent nomenclature review by prominent microglia experts suggested using state until a consensus is reached on specific microglial *subpopulation*, *subtype*, or *subset*^72^. When a known function is ascribed to a specific cellular state, we refer to this as a cellular *phenotype*. Any shift from one phenotype to another would be an example of *plasticity*. A further challenge in the microglial field is how to describe microglia that arise from distinct developmental origins—microglia can arise for the yolk sac or from early foetal monocytes^1^. Scientific consensus should in the future address these challenges^72^.

## How is microglia heterogeneity studied?

Microglia are routinely defined to be heterogenous^6–8,16,21–25^. What does this mean and how do researchers reach these conclusions? The most common tools used to define cells and their transcriptional states are scRNAseq and snRNAseq, although other high dimensional approaches such as cytometry by time of flight (CyTOF) or spectral flow cytometry are available. These experiments begin with sample collection, sample processing, sequencing, alignment to a select genome, and finally, computational analysis. Understanding the technical underpinnings of each step is fundamental to understanding how researchers conclude that a given microglial population is heterogeneous.

Single cell and nuclei approaches are widespread with the recent commercialization of the Chromium system by 10X genomics, as well as other options (detailed in Table 1). These approaches measure cDNA converted from mRNA that is amplified, fragmented, and then counted as a ‘read’ by a sequencing platform. Most of these approaches use a unique molecular identifier (UMI), a random sequence of nucleotides used to tag and count mRNA. The benefit of a UMI is that it corrects for PCR-induced artifacts during the library preparation steps^26^. The UMI also enables molecular counting, which is an advantage over previous approaches that normalized data to overall read counts. With so many different sc/snRNAseq platforms, it is important to consider the impact of method selection. Ding and colleagues compared seven of these methods, including two low throughput and five high throughput scRNAseq methods^27^. Major differences were found with respect to sensitivity, or the ability to capture RNA molecules as reflected by the average number of detected UMI or genes per cell. Low throughput methods had greater sensitivity than high throughput methods, with the most common system used to study microglia—the 10X Genomics Chromium platform—outperforming other high throughput approaches^27^. In a systematic comparison study that controlled for the bioinformatic pipeline, sensitivity differences affected the ability to identify distinct cell types when clustering peripheral blood mononuclear cell (PBMC) from scRNAseq data. Generally, more sensitive methods were better able to accurately define the percentage of a given cell population residing in the PBMC fraction. For microglia, the low throughput method Smart-seq2 and high throughput 10X Chromium platform were similarly able to detect an activated and white matter associated microglial state in the aged brain^25^.

**Table 1:** Overview of single-cell sequencing and preprocessing approaches.

Sequencing technique	Example study	Pre-processing	Example study
Chromium 10X genomics	^16^	Cell Ranger	^14^
inDrop	^60^	featureCounts	^61^
Drop-seq	^62^	UMI tools	^63^
Smart-seq2	^64^	RSEM	^65^
CEL-seq2	^66^	Kallisto	^67^
Microwell-Seq	^68^		
Seq-Well	^69^		
BDRhapsody system	^70^		
SPLiT-Seq	^71^		

An alternative approach to improve UMI and gene counts, is to increase the number of reads per cell, or sequencing depth. Like any estimate, increasing the number of objects counted—in this case, reads—reduces noise. Increased sequencing depth leads to reduced measurement noise^28–30^. An important question is, what is the optimal read depth to allow for the differentiation of cellular states? While it has not been addressed systematically with microglia, Pollen and colleagues addressed this directly by comparing high sequencing depth to lower depths and found that as few as 10,000–50,000 reads per cell was sufficient to group developing human neurons^29^. At lower sequencing coverage fewer genes were detected, the genes that were not captured were primarily genes expressed at low levels. Similarly, Heimberg and colleagues found that as few as 1000 transcripts could distinguish hippocampal pyramidal neurons from cortical pyramidal neurons^30^. However, they found that greater read depth provides a more accurate assessment of the variance, which may be important for defining more nuanced cellular states.

Following the sc/snRNAseq run, read or count data will undergo preprocessing via a number of pipelines for UMI or non-UMI based methods, detailed in Table 1. When Chen and colleagues benchmarked these across a range of samples and platforms, there was only modest variance in the number of genes detected per cell^31^. The chosen preprocessing pipeline used is therefore a minor contributor to gene detection variance. After prepossessing, it is common to normalize data. Normalization is a strategy to account for scRNAseq technical variation such as difference in read depth across cells or libraries. For example, scRNAseq data contains a high number of zero read counts^32^. While initially it was thought that scRNAseq data may be zero inflated, recent modelling with droplet scRNAseq suggests that the overabundance of zero values can be attributed to biological variation or challenges in measuring small numbers of transcripts at a single-cell level^33^. To account for the distribution of scRNAseq data, several normalization approaches are available. Chen and colleagues compared eight different normalization methods and found that six out of eight methods assessed preformed equivalently well including sctransform, scran deconvolution, CPM, DEseq, and Linnorm^31^.

When comparing common scRNAseq pipelines the most impactful factor was correcting for batch effects, or the stochastic effects imposed on different samples prepared at different times. Batch correction is especially important for low throughput approaches because often many libraries are collected to obtain enough cells. Chen and colleagues found large disparities in batch correction approaches^31^. Some batch correction methods performed well in accounting for biologically similar samples, or shared subpopulations between samples. Other approaches were useful for integrating samples with distinct cell types. Therefore, batch correction does not appear to be a one size fits all approach and researchers should tailor the batch correction tool to the sample they are integrating. For many of the landmark papers using single-cell RNA sequencing to characterize microglial states, researchers use low throughput approaches where often hundreds of libraries are required to obtain enough cells^6,7,23,24,34–36^. How batch effect and batch correction alters the interpretation of the studies is rarely reported.

## How is microglia heterogeneity assessed?

Following preprocessing, normalization, and integration, researchers will cluster their data and project it onto a t-Distributed Stochastic Neighbor Embedding (TSNE), or Uniform Manifold Approximation and Projection (UMAP) plot^37^. To define microglia heterogeneity, researchers use two main clustering algorithms: k-means clustering^9,23,35,38^ or community assessment with Louvain algorithm^6,7,16,39–41^. For k-means clustering, the number of clusters (k) are chosen by the user before the data is iteratively clustered around *k* clustering centres, with each cell assigned to its closest cluster centre using Llyod’s Algorithm^42^. However, this biases clusters towards those of equal sizes and, as a result, rare populations can be missed. Therefore, a package that uses k-means clustering, called RaceID, introduced an outlier detection method to counteract this issue^43^. Community-based detection algorithms like Louvain’s algorithm, detects clusters based on ‘communities’, which is the basis for the popular scRNAseq tool, Seurat^44^ used by many microglial researchers^6,7,16,25,34,39–41,45–47^. Either clustering algorithm separates cells based on similarities and the proximity of each cell within the graphical projection indicates their relatedness. Clusters can be defined or annotated using knowledge of the sample collection, previously identified gene-expression profiles, or available automated annotation software (reviewed by^48^). Given that most labs use one clustering algorithm, it is not yet clear how the choice of clustering algorithm alters our understanding of cellular heterogeneity.

**Fig. 1:**
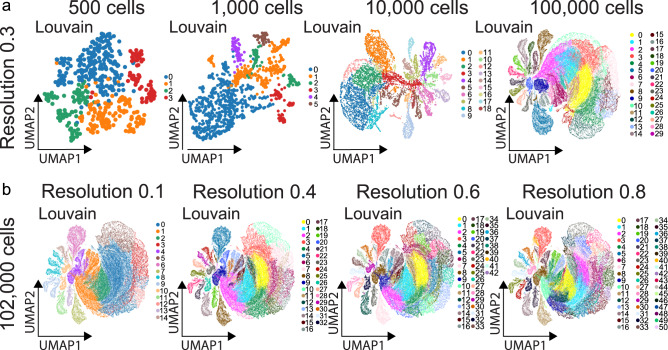
Number of cells and bioinformatic settings impact cluster numbers. Astrocyte transcriptional data was obtained from the publicly available mouse brain dataset (https://support.10xgenomics.com/single-cell-gene-expression/datasets)^57^. We chose to use astrocyte data as an exemplar due to the availability of a sufficiently comprehensive and large dataset. Cells were clustered using the Louvain algorithm in Scanpy^44,58^. **a** Data was optimized using the machine learning algorithm SCCAF^59^ and data was projected onto a UMAP. By increasing the cell number analyzed through subsampling of original data (from 500, 1000, 10,000, or 100,000 cells) there is an increase in the number of clusters under similar analytic conditions. Analyzing more cells, therefore, yields more clusters. **b** Clustering a similar number of cells (102,000 astrocytes) using Louvain’s algorithm while varying resolution demonstrates that a higher resolution produces more clusters.

Two chief strategies are emerging to define microglia heterogeneity: low throughput and high throughput approaches. Many researchers opt for a low throughput approach, which typically collects as much as 30–40 percent of the mRNA molecules in a cell^49^. However, with low throughput approaches many hundreds or even thousands of libraries need to be collected, requiring the application of imperfect integration or batch correction strategies^31^. An alternative strategy is to use a high throughput approach to collect thousands of cells in a single library^50^. However, with higher throughput only 5–20 percent of mRNA molecules are collected, resulting in fewer genes detected per cell^27^. What is clear is that understanding cellular heterogeneity requires the capture of large numbers of cells. Using astrocytes collected from a commercially available source we show how more cells results in greater clarity of astrocyte clusters, with nearly four times more clusters identified with 10,000 cells than 1000 cells (Fig. 1). However, clustering does not carry inherent biological meaning. Indeed, one adjustable bioinformatic attribute known as ‘resolution’ can alter the number of clusters identified (Fig. 1). The method of resolution selection depends on the type of clustering analysis used. While strategies for the selection of an optimal resolution are available^37,51^, decisions about the level of resolution remain subjective.

Obtaining a high number of cells is important to detect small populations, but can also amplify noise. For example, cells die during dissociation and not all of these dying cells will be excluded with quality control. These damaged cells may form their own cluster if their numbers are high enough, which is unlikely to be a functional subpopulation. Similarly, doublet or multiplet cells are not their own state. Quality control measures during the bioinformatic pipeline such as mitochondrial gene counts and total gene counts are meant to limit these confounding populations, but they may still be present in small numbers after quality control measures. Another concern is dissociation artifacts, or changes in a cell population based on how the cells are dissociated. Fortunately, using transcriptional and translational inhibitors researchers minimize ex vivo dissociations artifacts^52^. Taken together, it is important to validate an identified microglial states by using orthogonal approaches. Validation of cellular heterogeneity can be done with immunohistochemistry or fluorescent in situ hybridization (FISH) approaches such as seqFISH^53^, RNAscope^54^, and merFISH^55^. Bioinformatic approaches, such as gene regulatory network assessment using tools like Single-cell Regulatory Network Interference and Clustering (SCENIC)^56^, increase confidence that a cluster is biologically relevant and not a consequence of the clustering technique applied to the data.

## Concluding remarks

Semantic satiation was coined by Leon Jakobovits James to describe the phenomenon in which a word temporarily loses its meaning when listened to, verbalised, or read repeatedly over a short period of time. In this era of multi-omics, the word heterogeneous is one of the most frequently used words to describe microglia. Given its high frequency of use, we feel compelled to suggest a cautious approach to its interpretation. When examined more carefully, this word can be used to describe a wide range of experimental results, with the interpretation left up to the reader. We believe that the word heterogeneity should at the very least be defined and not act as a conclusion. For example, the results for a single-cell RNA sequencing study would find heterogeneous cellular states. Future work may then identify functional heterogeneity. Ultimately, reports of microglia heterogeneity on a transcriptional level have generated huge interest, primarily due to the inference that transcriptional heterogeneity begets functional heterogeneity. Future work is still required to elucidate functional differences between these diverse microglial states with the hope that these observations will aid our understanding of the roles microglia play in disease and injury. A clearer understanding of microglia heterogeneity will pave the way for therapeutic interventions that target specific phenotypes or subset(s) of microglia while leaving the bulk of the brain’s microglia population unaffected.

### Reporting summary

Further information on research design is available in the Nature Research Reporting Summary linked to this article.

## Supplementary information

Reporting Summary

## Data Availability

Data is available from (https://support.10xgenomics.com/single-cell-gene-expression/datasets) called “1.3 million brain cells from E18 mice” or NCBI GEO as (GSE93421)^57^.

## Code availability

The code we used is all publicly available from other research groups^44,58,59^.
